# Offset of openings in optic nerve head canal at level of Bruch’s membrane, anterior sclera, and lamina cribrosa

**DOI:** 10.1038/s41598-021-01184-8

**Published:** 2021-11-17

**Authors:** Kyoung Min Lee, Hyoung Jun Ahn, Martha Kim, Sohee Oh, Seok Hwan Kim

**Affiliations:** 1 Department of Ophthalmology, Seoul National University College of Medicine, Seoul, Korea; 2 Department of Ophthalmology, Seoul National University Boramae Medical Center, 39 Boramae Road, Dongjak-gu, Seoul, 07061 Korea; 3 Department of Mathematical Modeling, Mind Flow Lab, Seoul, Korea; 4 Department of Ophthalmology, Dongguk University Ilsan Hospital, Goyang, Korea; 5 Department of Biostatistics, Seoul National University Boramae Medical Center, Seoul, Korea

**Keywords:** Anatomy, Neurology, Mathematics and computing

## Abstract

We compared the central retinal vascular trunk (CRVT) position, as a surrogate of lamina cribrosa (LC) offset, with the anterior scleral opening (ASCO) offset from the Bruch’s membrane opening (BMO). Based on the BMO-centered radial scans, the BMO and ASCO margins were demarcated, and each center was determined as the center of the best-fitted ellipse for each margin. The ASCO/BMO offset was defined as the offset between each center. Angular deviations and the extent of ASCO and CRVT offsets from the BMO center were compared directly. Incomplete demarcation of ASCO was found in 20%, which was associated with a larger BMO area and a larger ASCO offset from the BMO. The angular deviation of ASCO offset was associated with that of CRVT offset and that of the longest externally oblique border. The ASCO offset was smaller than the CRVT offset, and, unlike the CRVT offset, it was rarely deviated to the inferior side. The complete ASCO margin might not be demarcatable when determined on BMO-centered radial scans in the presence of an offset. Also, the ASCO, which reflects only the superficial scleral layer, might not reflect the LC position, because the LC might be shifted further from the ASCO.

## Introduction

**Figure 1:**
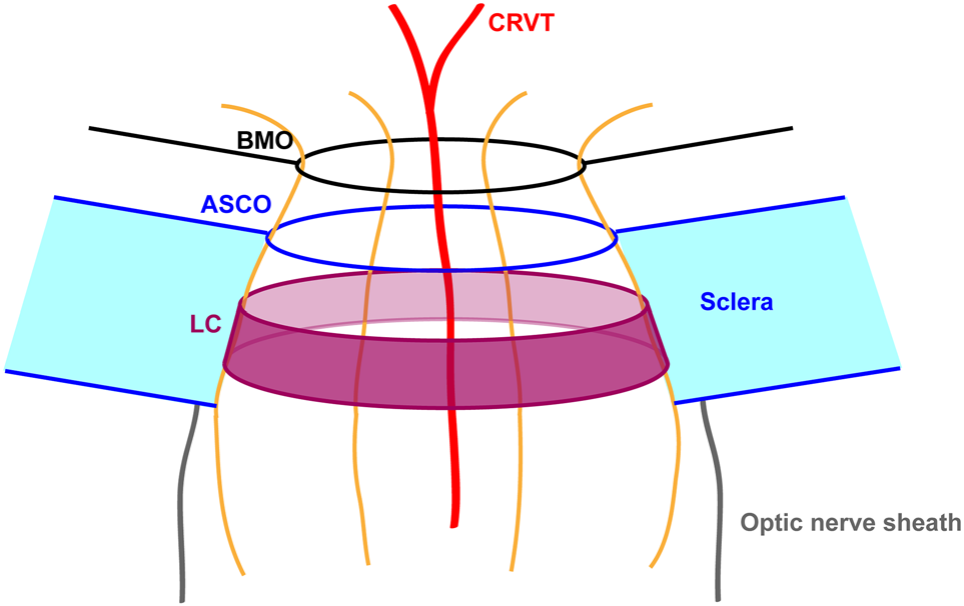
Schematic drawing of three openings in optic nerve head (ONH) canal. The black lines indicate the Bruch’s membrane (BM) with central opening (BMO). The blue lines indicate the sclera and its anterior opening, defined as the anterior scleral opening (ASCO; blue circle). The lamina cribrosa (LC; purple cylinder) is located at a lower level than the ASCO is. The axons passing through the ONH canal are drawn as yellow fibers. The central retinal vascular trunk (CRVT; red line) also passes through the ONH canal. The optic nerve sheath (gray line) is anchored to the posterior sclera near the ONH canal. The alignments among the BMO, ASCO, and LC are known to change during eyeball expansion. This study was designed to compare the ASCO and CRVT (a surrogate of LC position) offsets from the BMO.

The optic nerve head (ONH) is a complex structure that guides the retinal ganglion cell (RGC) axons from inside to outside of the eyeball through a canal. This ONH canal faces three openings by three encountered layers (Fig. 1): (1) the retinal layer–the Bruch’s membrane opening (BMO), (2) the anterior scleral layer–the anterior scleral opening (ASCO), and (3) the lamina cribrosa (LC). These openings, however, are known to be imperfectly aligned from end to end^1–3^. The offset between the BMO and ASCO can be observed as a sign of parapapillary atrophy (PPA; γ-zone) on enface images or as the externally oblique border (EOB) on cross-sectional images^4–8^. Interestingly, these PPA or EOB are associated with the preferential site of RGC axonal loss: glaucomatous optic neuropathy^9–13^.

The Boramae Myopia Cohort Study revealed that offset is incurred by divergent retinal and scleral layer growth during eyeball expansion^14–16^. Upon expansion, the retina might relatively preserve the posterior polar structure (including BMO) by preferential growth in the equatorial area, while the outer-load bearing scleral structure (including LC) expands, which discrepancy can lead to a relative shift of the sclera and LC from the BMO^14–17^. Shift and consequent offset of LC is clinically important, since it can explain the association of all these changes with the preferential site of glaucomatous damage^18,19^.

In those studies, the LC offset was estimated with reference to that of the central retinal vascular trunk (CRVT)^14–17^, because the CRVT is embedded in the dense connective tissue of the LC^20^ and is located in the center of ONH in most of newborns^21,22^. Recently, ASCO/BMO offset has been studied^23,24^. If the ASCO margin can be demarcated completely and the ASCO offset from the BMO represents the LC offset, the ASCO/BMO offset might be used as a better surrogate for LC offset. To the best of our knowledge, there has been no study comparing ASCO and LC offsets from the BMO in order to evaluate ONH outer-wall shift. The purpose of this study, then, was to compare ASCO center offset and CRVT offset from the BMO center, as representative of ASCO/BMO and LC/BMO offsets, respectively.

## Results

One hundred and sixteen (116) eyes of 116 participants were recruited between January 2019 and December 2020. Of these, 6 subjects were excluded due to incomplete ASCO demarcation for more than 90° continuously, 2 subjects due to incomplete BMO demarcation, 1 due to poor spectral-domain (SD) optical coherence tomography (OCT) image quality, and 1 due to bifurcation of the CRVT, which resulted in a final sample of 106 eyes of 106 subjects (53 open-angle glaucoma eyes and 53 eyes without glaucoma). In 10 eyes, emergence of CRVT was not visible, and its absence within the BMO was proved using OCT angiography. The subjects were aged 56.5 ± 13.6 years, and had a refractive error of -2.60 ± 2.99 diopters, and an axial length of 25.1 ± 1.5 mm; 46 of the subjects (43%) were female (Table 1).

**Table 1:** Demographic data according to completeness of anterior scleral opening (ASCO) demarcation.

	Complete demarcation (N = 85)	Incomplete demarcation (N = 21)	*P*
Age, years	58.8 ± 12.5	46.9 ± 13.8	< 0.001*
Sex (Male/Female)	47/38	13/8	0.584^†^
Axial length, mm	24.8 ± 1.5	26.1 ± 1.2	< 0.001^‡^
IOP, mmHg	13.7 ± 2.3	14.2 ± 2.9	0.358*
BMO area, mm^2^	2.62 ± 0.89	3.13 ± 0.67	0.001^‡^
Foveal-BMO axis, °	− 7.0 ± 4.4	− 5.9 ± 3.3	0.513^‡^
ASCO area, mm^2^	2.61 ± 0.58	2.74 ± 0.59	0.112^‡^
Shift Index	0.36 ± 0.21	0.75 ± 0.25	< 0.001^‡^
Offset ratio (CRVT/ASCO)	2.30 ± 2.19	1.70 ± 0.58	0.783^‡§^
Glaucoma	43 (51%)	10 (48%)	0.807^†^

IOP, Intraocular pressure; BMO, Bruch’s membrane opening; ASCO, Anterior scleral canal opening; CRVT, Central retinal vascular trunk.

*Comparison performed using independent-*t* test.

^†^Comparison performed using Chi-square test.

^‡^Comparison performed using Mann–Whitney U test.

^§^Eyes with shift index = 1.0 were excluded, since we could not precisely determine the extent of CRVT offset.

The reproducibility of results for determination of the centers of the BMO & ASCO, the CRVT location, and the meridian of the longest EOB was evaluated based on the *x* and *y* coordinate values of the pixels obtained by two independent observers^25,26^. The interobserver reliability was excellent for the BMO center, the ASCO center, the CRVT location, and for the meridian of the longest EOB (Supplemental Table 1).

**Figure 2:**
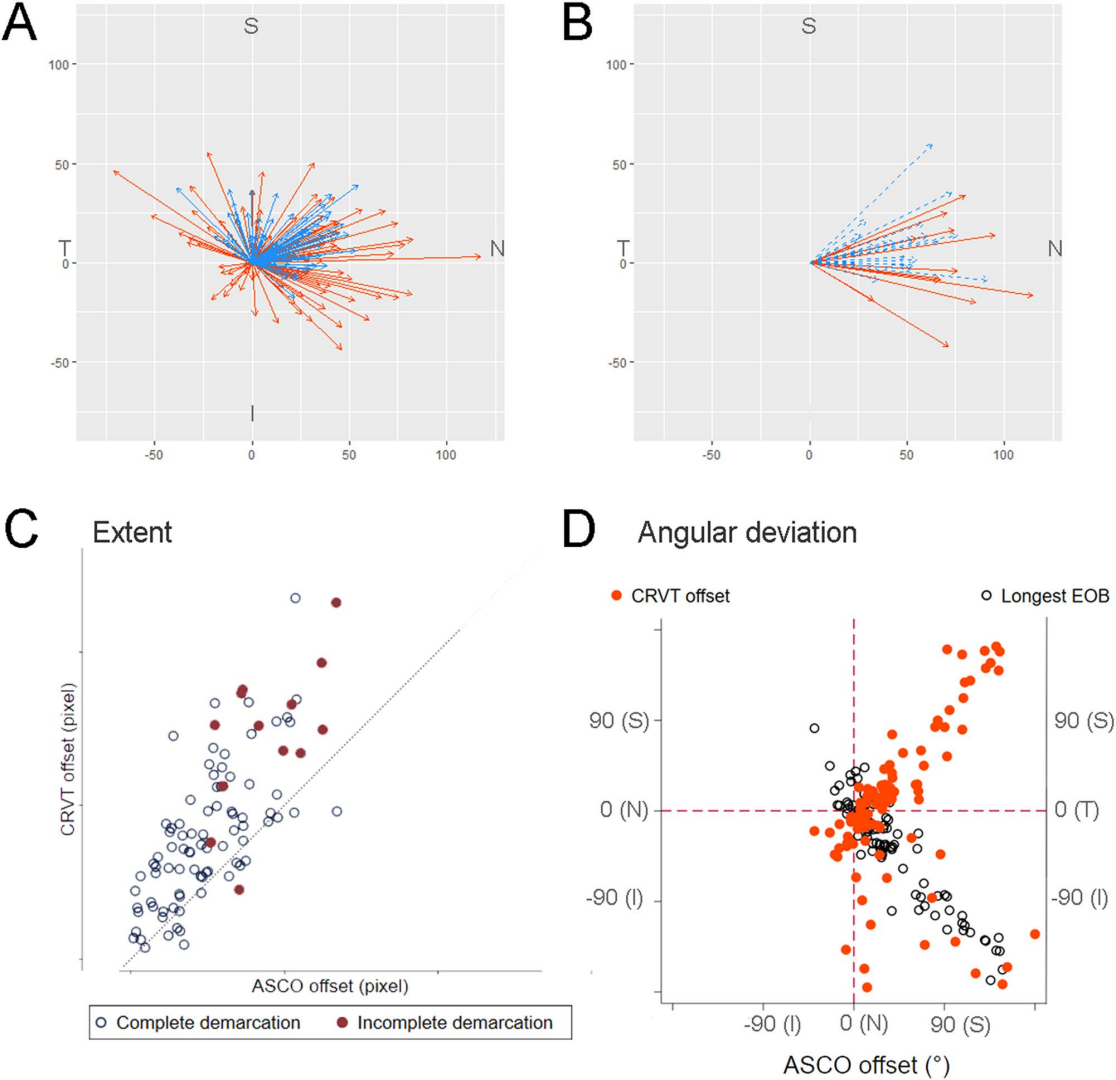
Offsets of anterior scleral opening (ASCO), central retinal vascular trunk (CRVT), and longest externally oblique border (EOB). (**A**, **B**) Vector fields comparing the CRVT (orange arrows) and the ASCO offsets (blue arrows) in eyes with complete ASCO demarcation (**A**) and in eyes with incomplete ASCO demarcation (**B**). Both offsets share directionalities, while extent is larger for CRVT offset than for ASCO offset. Please note that the eyes with the largest CRVT offset (shift index = 1.0) are not even drawn in the vector fields because we could not define the directionality for the CRVT offset in those cases. (**C**) Scatter plot comparing extent of CRVT and ASCO offsets within individuals by pixels. Regardless of complete or incomplete ASCO demarcation, CRVT offset tends to be larger than ASCO offset. The eyes with the largest CRVT offset (shift index = 1.0) were excluded from the analysis because we could not measure the exact pixel values of CRVT offset in those cases. (**D**) Scatter plot comparing angular deviations of CRVT offset, ASCO offset, and longest EOB. Please note that ASCO offset is barely deviated inferiorly. S = superior; T = temporal; I = inferior; N = nasal.

**Figure 3:**
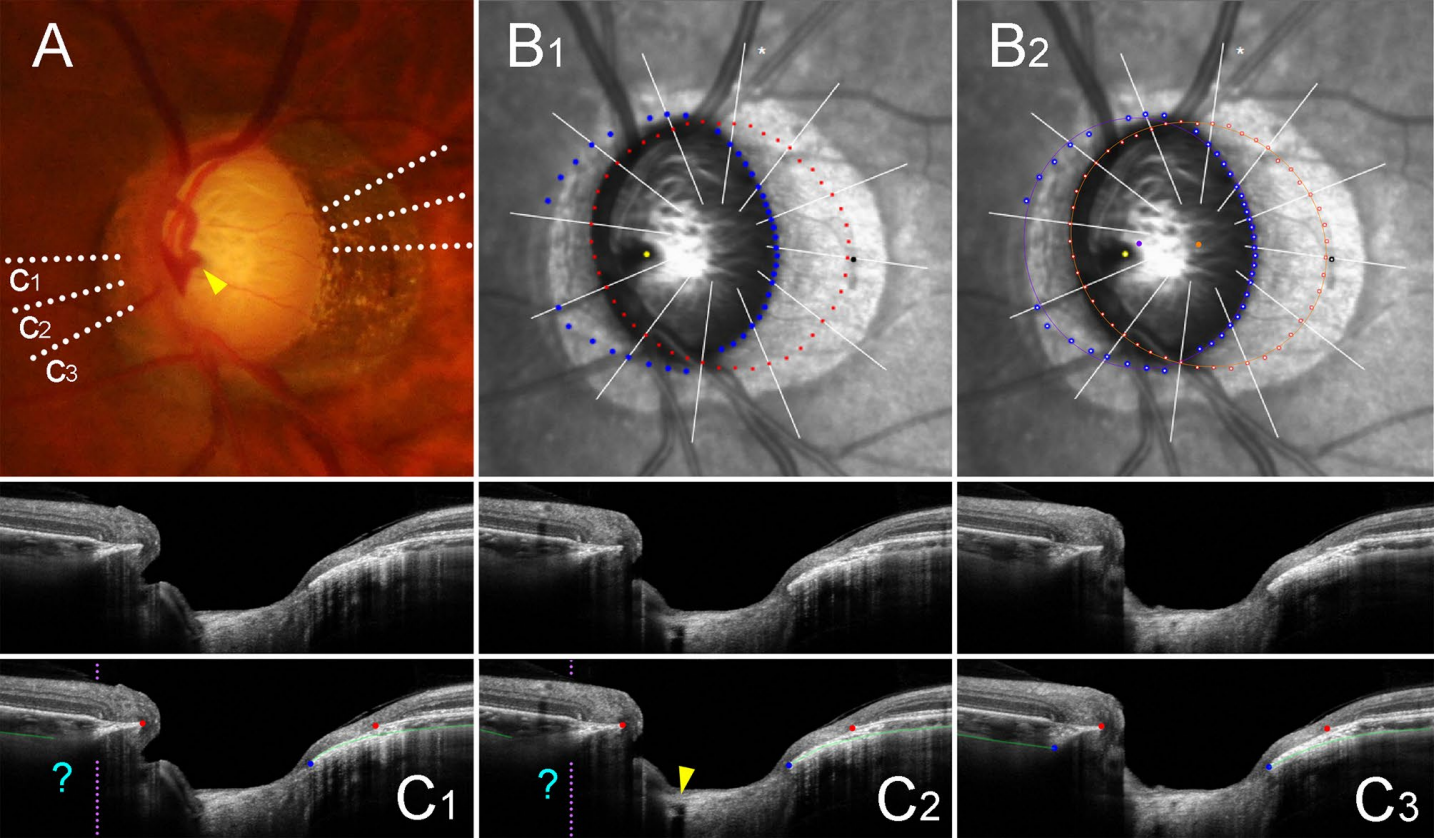
Sample case of outer-wall shift to nasal side (left eye of 47-year-old man with glaucoma, axial length of 26.68 mm). (**A**) Disc photograph. The arrowhead indicates the location of the central retinal vascular trunk (CRVT). The dotted lines indicate the location of the OCT scan. (**B**) Infrared images. (**B**_**1**_) Bruch’s membrane opening (BMO, red dots), anterior scleral opening (ASCO, blue dots), CRVT position (yellow dot), and meridian of longest externally oblique border (EOB, black dot) are marked. (**B**_**2**_) Best-fitted ellipses for BMO (orange ellipse) and ASCO (purple ellipse) are drawn with their centers (orange dot for BMO center, purple dot for ASCO center). (**C**) B-scan OCT images (top row: original images, bottom row: images with labels). The red dots indicate the BMO margin; the blue dots indicate the ASCO margin; the green lines indicate the anterior scleral surface; the arrowhead indicates the CRVT. On the nasal side, the ASCO margin cannot be drawn (**C**_**1**_, **C**_**2**_**,** question mark). The ASCO margin can be drawn on the adjacent scan (**C**_**3**_). Since the indiscernible angle is larger than 30° on the nasal side, this eye is classified as incomplete demarcation for ASCO and grouped accordingly. Despite the incomplete ASCO demarcation, the best-fitted ellipse could be fitted, and the boundary according to the fitting is marked by the purple dotted lines (**C**_**1**_, **C**_**2**_). Please note that both the CRVT and ASCO offsets are headed nasally, while the extent is larger for the CRVT offset than for the ASCO offset.

**Figure 4:**
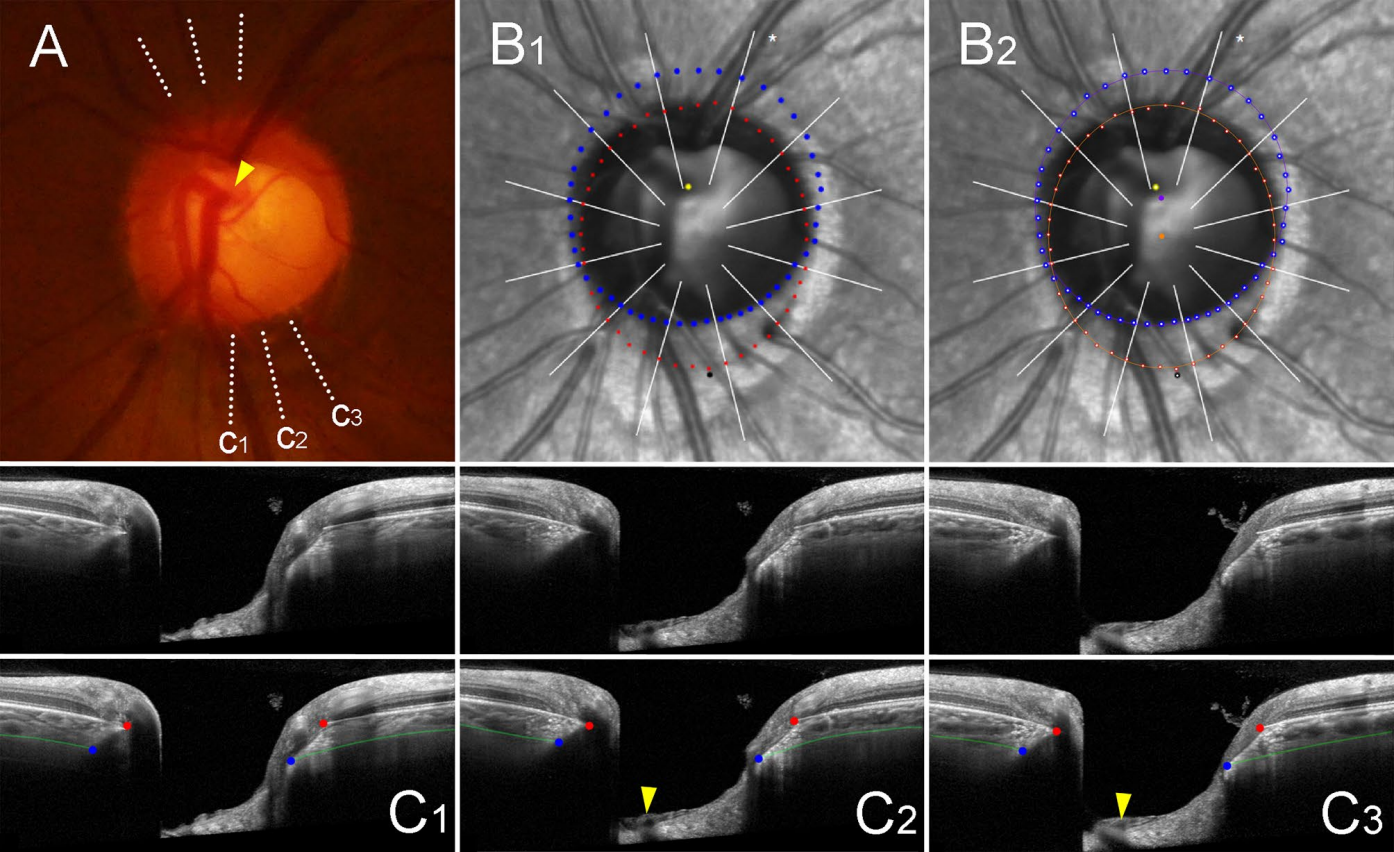
Sample case of outer-wall shift to superior side (left eye of 44-year-old man with glaucoma, axial length of 25.36 mm). (**A**) Disc photograph. The arrowhead indicates the location of the central retinal vascular trunk (CRVT). The dotted lines indicate the location of the OCT scan. (**B**) Infrared images. (**B**_**1**_) Bruch’s membrane opening (BMO, red dots), anterior scleral opening (ASCO, blue dots), CRVT position (yellow dot), and meridian of longest externally oblique border (EOB, black dot) are marked. (**B**_**2**_) Best-fitted ellipses for BMO (orange ellipse) and ASCO (purple ellipse) are drawn with their centers (orange dot for BMO center, purple dot for ASCO center). (**C**) B-scan OCT images (top row: original images, bottom row: images with labels). The red dots indicate the BMO margin; the blue dots indicate ASCO margin; the green lines indicate the anterior scleral surface; the arrowhead indicates the CRVT. The ASCO margin is completely demarcatable, and thus, this eye is classified into the complete demarcation group for ASCO. Please note that both the CRVT and ASCO offsets are headed superiorly, while the extent is larger for the CRVT offset than for the ASCO offset.

**Figure 5:**
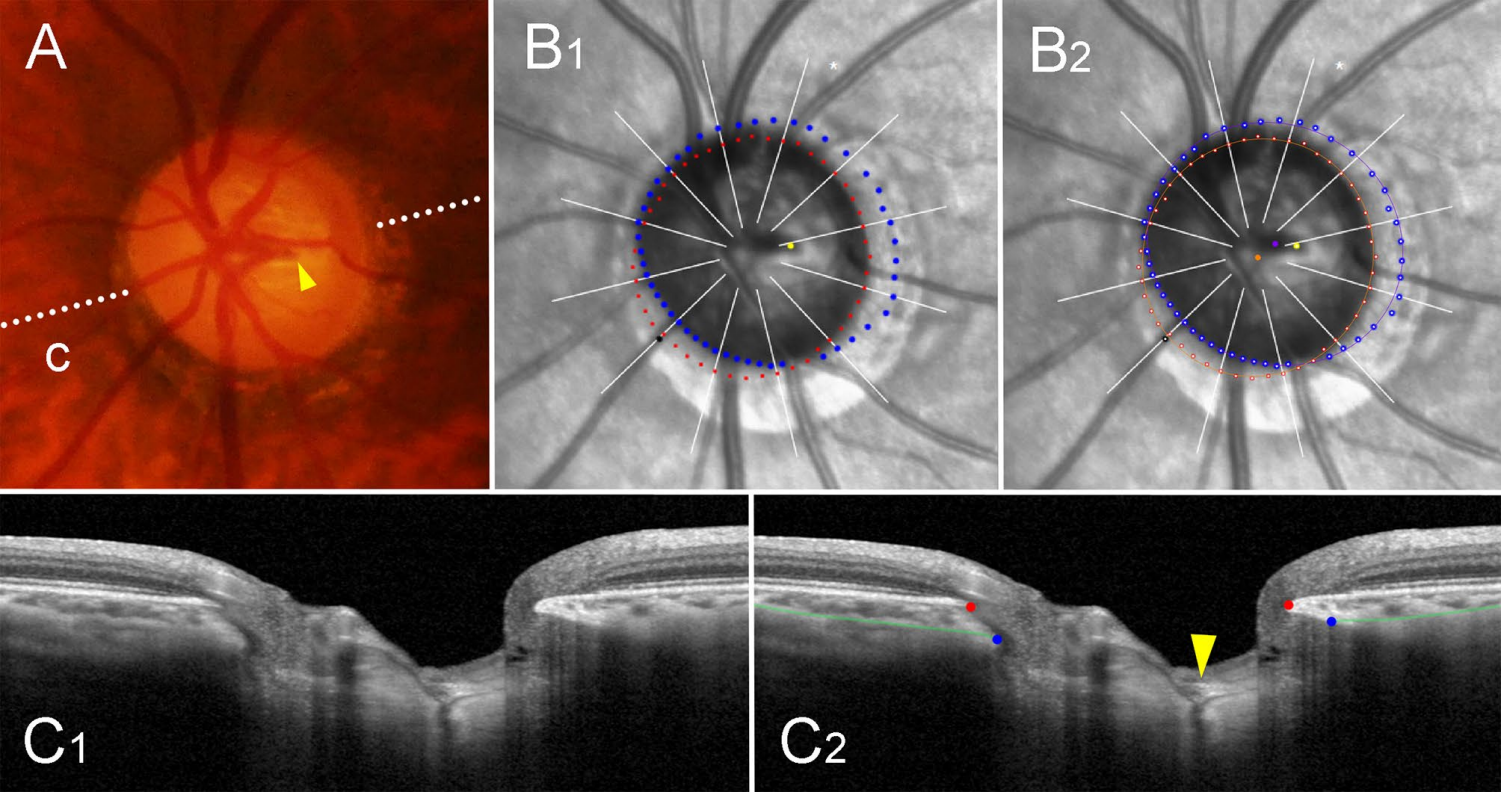
Sample case of outer-wall shift to temporal side (left eye of 59-year-old woman with glaucoma, axial length of 26.35 mm). (**A**) Disc photograph. The arrowhead indicates the location of the central retinal vascular trunk (CRVT). The dotted lines indicate the location of the OCT scan. (**B**) Infrared images. (**B**_**1**_) Bruch’s membrane opening (BMO, red dots), anterior scleral opening (ASCO, blue dots), CRVT position (yellow dot), and meridian of longest externally oblique border (EOB, black dot) are marked. (**B**_**2**_) Best-fitted ellipses for BMO (orange ellipse) and ASCO (purple ellipse) are drawn with their centers (orange dot for BMO center, purple dot for ASCO center). (**C**) B-scan OCT images. (**C**_**1**_) original image. (**C**_**2**_) image with labels, which clearly shows emergence of CRVT (arrowhead). The green lines indicate the anterior scleral surface. Please note that both the CRVT and ASCO offsets are headed temporally, while the extent is larger for the CRVT offset than for the ASCO offset.

**Figure 6:**
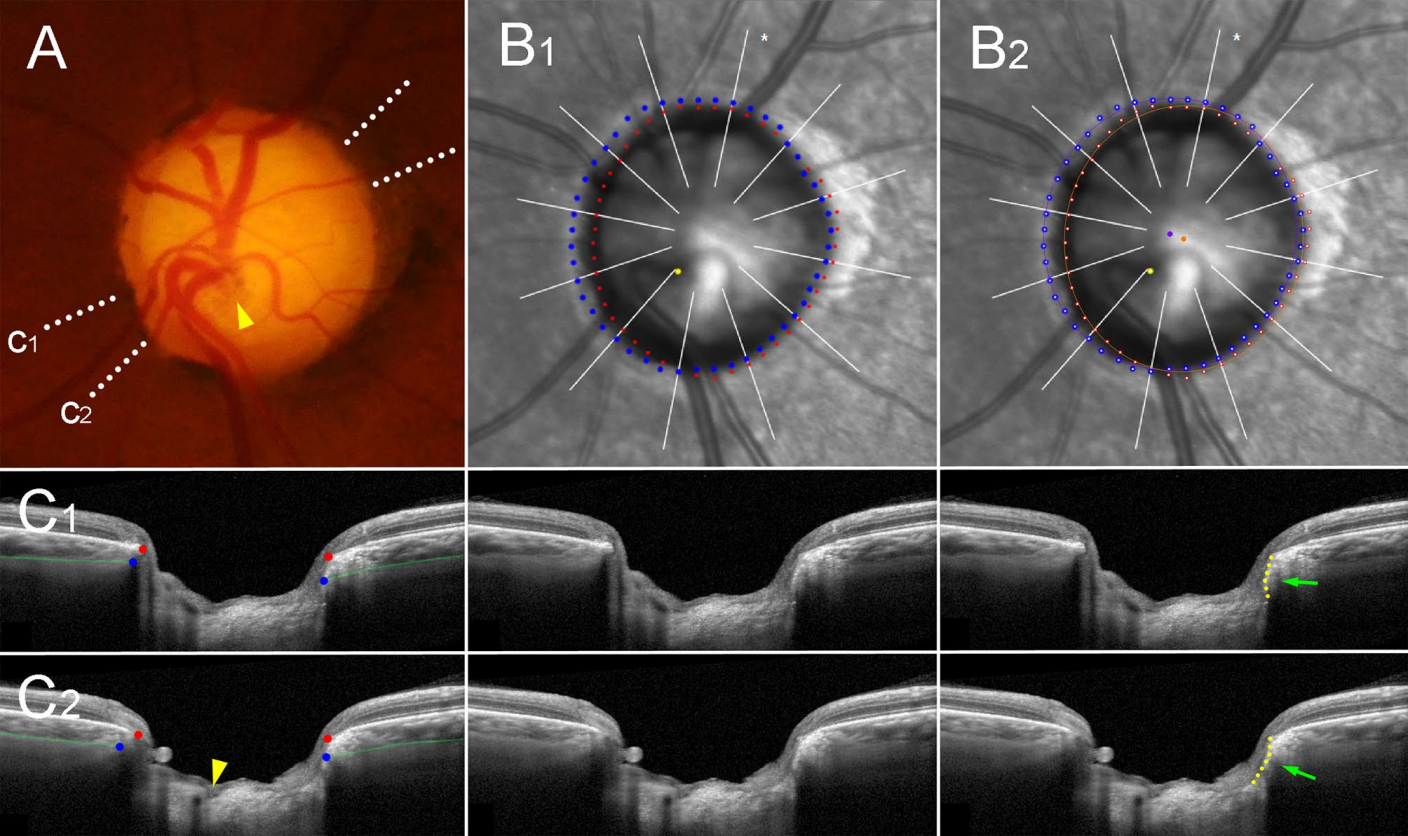
Sample case of outer-wall shift to inferonasal side (left eye of 58-year-old woman with glaucoma, axial length of 23.94 mm). (**A**) Disc photograph. The arrowhead indicates the location of the central retinal vascular trunk (CRVT). The dotted lines indicate the location of the OCT scan. (**B**) Infrared images. (**B**_**1**_) Bruch’s membrane opening (BMO, red dots), anterior scleral opening (ASCO, blue dots), and CRVT position (yellow dot) are marked. (**B**_**2**_) Best-fitted ellipses for BMO (orange ellipse) and ASCO (purple ellipse) are drawn with their centers (orange dot for BMO center, purple dot for ASCO center). (**C**) B-scan OCT images. The left column shows OCT images with demarcation of the BMO margin (red dots) and ASCO margin (blue dots). The green lines indicate the anterior scleral surface. The arrowhead indicates the CRVT. The middle column shows unlabeled OCT images. The right column shows OCT images with demarcation of the peripapillary border tissue of the choroid (PBT-C, upper part of yellow dotted lines) and that of the scleral flange (PBT-S, lower part of yellow dotted lines). The green arrows indicate the ASCO margin, which connects the PBT-C and PBT-S. Please note that the outer-wall shift to the nasal (**C**_**1**_) and inferior (**C**_**2**_) sides is more severe in the more-outer layer (the lamina cribrosa [LC] with CRVT) than in the superficial layer (ASCO), and is presented as an abrupt change between the PBT-C and the PBT-S. This could explain why the CRVT alone is deviated while the ASCO is immediately next to the BMO in this case.

From the BMO center, the angular deviation of the ASCO offset shared same directionalities with that of the CRVT offset (*r* = 0.342, *P* < 0.001; Fig. 2). The distance was longer for the CRVT offset than for the ASCO offset (*P* < 0.001, paired *t*-test; Fig. 2C); the offset ratio (CRVT/ASCO) was 2.22 ± 2.06 (95% confidence interval 1.80–2.63), even after excluding 10 eyes with the largest CRVT offset (shift index = 1.0). ASCO offset to the nasal side (offset angle between -45° and 45°) was associated with CRVT offset to the further nasal side (Fig. 3), with the offset ratio of 1.96 ± 1.18 (95% confidence interval 1.66–2.26). ASCO offset to the superior side (offset angle between 45° and 135°) was associated with CRVT offset to the further superior side (Fig. 4), with the offset ratio of 2.70 ± 3.29 (95% confidence interval 1.37–4.03). ASCO offset to the temporal side (offset angle larger than 135° or smaller than -135°) was also associated with CRVT offset to the further temporal side (Fig. 5), with the offset ratio of 2.74 ± 2.34 (95% confidence interval 0.58–4.91). No eyes had the ASCO offset to the inferior side (offset angle between -45° and -135°), whereas some eyes had the CRVT offset to the inferior side (Fig. 6). The offset ratio was larger than 1 in the control (2.42 ± 2.57 [95% confidence interval 1.69–3.15]), glaucoma (1.99 ± 1.29 [95% confidence interval 1.61–2.37]), and myopia groups (1.88 ± 1.24 [95% confidence interval 1.57–2.19]) when analyzed separately. Multiple regression analysis revealed that the angular deviation of the CRVT offset (*P* < 0.001) and that of the ASCO offset (*P* = 0.015) were both associated with the offset ratio (Table 2).

**Table 2:** Factors associated with offset ratio: central retinal vascular trunk (CRVT) offset divided by anterior scleral canal opening (ASCO) offset.

	Univariable analysis			Multivariable analysis*		
	Coefficient	95% CI	*P*	Coefficient	95% CI	*P*
Age, years	0.019	(− 0.014, 0.051)	0.256			
Female (vs. male sex)	− 0.092	(− 0.939, 0.756)	0.831			
Axial length, mm	− 0.332	(− 0.604, − 0.061)	0.017	− 0.103	(− 0.383, 0.176)	0.464
IOP, mmHg	0.038	(− 0.145, 0.222)	0.678			
BMO area, mm^2^	− 0.592	(− 1.061, − 0.123)	0.014	− 0.243	(− 0.697, 0.211)	0.290
Foveal-BMO axis, °	0.010	(− 0.089, 0.108)	0.846			
ASCO area, mm^2^	− 0.449	(− 1.175, 0.277)	0.223			
**CRVT offset**, °	− **0.001**	**(**− **0.015,** − **0.005)**	** < 0.001**	− **0.012**	**(**− **0.017,** − **0.007)**	** < 0.001**
**ASCO offset**, °	**0.008**	**(**− **0.001, 0.017)**	**0.065**	**0.012**	**(0.002, 0.022)**	**0.015**
Meridian of longest EOB, °	− 0.000	(− 0.004, 0.003)	0.896			
Shift index	− 0.912	(− 2.910, 1.086)	0.367			
Glaucoma	− 0.436	(− 1.271, 0.399)	0.303			

Statistically significant values (*P* < 0.05) are shown in bold.

CI, Confidence interval; IOP, Intraocular pressure; BMO, Bruch’s membrane opening; ASCO, Anterior scleral canal opening; CRVT, Central retinal vascular trunk; EOB, Externally oblique border.

*Variables with *P* < 0.10 in the univariable analysis were included in the subsequent multivariable analysis.

The angular deviation of ASCO offset also showed significant correlations with that of the longest EOB (*r* = − 0.933, *P* < 0.001). If we compared the absolute value of correlation coefficient to handle the opposite direction of reference meridian (the nasal horizontal midline was set to zero for the ASCO and CRVT offsets, while the temporal horizontal midline was set to zero for the longest EOB), the angular deviation of ASCO offset showed more correlation with that of the longest EOB than that of CRVT offset (*P* < 0.001, Williams’ test)^27^. In contrast to the angular deviations of the longest EOB and the CRVT offset, that of ASCO offset was rarely shifted to the inferior side (Fig. 2D). The multiple regression analysis revealed that the axial length (*P* = 0.003), the angular deviation of the CRVT offset (*P* = 0.002), and the meridian of the longest EOB (*P* < 0.001) were associated with the angular deviation of ASCO offset (Table 3). The association with the meridian of the longest EOB remained significant in the control, glaucoma, and myopia groups when analyzed separately (Supplemental Table 2).

**Table 3:** Factors associated with angular deviation of anterior scleral opening (ASCO) offset.

	Univariable analysis			Multivariable analysis*		
	Coefficient	95% CI	*P*	Coefficient	95% CI	*P*
Age, years	1.306	(0.679, 1.932)	< 0.001	− 0.082	(− 0.396, 0.232)	0.604
Female (vs. male sex)	37.808	(20.942, 54.675)	< 0.001	− 4.740	(− 13.390, 3.911)	0.278
**Axial length, mm**	− **13.909**	**(**− **19.374,** − **8.445)**	** < 0.001**	− **4.444**	**(**− **7.305,** − **1.583)**	**0.003**
IOP, mmHg	− 2.851	(− 6.643, 0.941)	0.139			
BMO area, mm^2^	− 14.059	(− 24.223, − 3.895)	0.007	3.330	(− 2.896, 9.556)	0.290
Foveal-BMO axis, °	− 2.895	(− 5.002, − 0.787)	0.008	− 0.628	(− 1.463, 0.208)	0.138
ASCO area, mm^2^	− 17.927	(− 33.312, − 2.542)	0.023	− 4.780	(− 14.113, 4.553)	0.311
**CRVT offset**	**0.216**	**(0.095, 0.338)**	**0.001**	**0.108**	**(0.040, 0.175)**	**0.002**
**Meridian of longest EOB**, °	− **0.804**	**(**− **0.869,** − **0.739)**	** < 0.001**	− **0.682**	**(**− **0.780,** − **0.583)**	** < 0.001**
Glaucoma	1.094	(− 17.143, 19.331)	0.906			

Statistically significant values (*P* < 0.05) are shown in bold.

CI, Confidence interval; IOP, Intraocular pressure; BMO, Bruch’s membrane opening; ASCO, Anterior scleral canal opening; CRVT, Central retinal vascular trunk; EOB, Externally oblique border.

*Variables with *P* < 0.10 in the univariable analysis were included in the subsequent multivariable analysis.

The eyes were classified according to the completeness of ASCO demarcation: (1) group 1, complete demarcation (85 eyes) and (2) group 2, incomplete demarcation (21 eyes; Table 1). This grouping showed excellent inter-observer reproducibility [Kappa statistic = 0.872 (95% confidence interval 0.748–0.997)]. Group 2 was of younger age and had a longer axial length, a larger BMO area, and a larger shift index relative to group 1 (Table 1). The logistic regression analysis revealed that incomplete demarcation of ASCO margin (group 2) was associated with larger BMO area (*P* = 0.036) and larger shift index (*P* < 0.001; Table 4). Larger shift index was also associated with incomplete ASCO demarcation in the control, glaucoma, and myopia groups when analyzed separately (Supplemental Table 3).

**Table 4:** Risk factors for incomplete demarcation of anterior scleral opening (ASCO) margin.

	Univariable analysis			Multivariable analysis*		
	OR	95% CI	*P*	OR	95% CI	*P*
Age, years	0.929	(0.890, 0.970)	0.001	0.989	(0.936, 1.046)	0.710
Female (vs. male sex)	0.761	(0.286, 2.026)	0.585			
Axial length, mm	1.925	(1.300, 2.850)	0.001	1.353	(0.837, 2.189)	0.217
IOP, mmHg	1.099	(0.900, 1.343)	0.356			
**BMO area, mm**^**2**^	**1.799**	**(1.053, 3.075)**	**0.032**	**2.082**	**(1.050, 4.130)**	**0.036**
Foveal-BMO axis, °	1.070	(0.941, 1.218)	0.301			
ASCO area, mm^2^	1.445	(0.647, 3.229)	0.369			
**Shift Index**	**513.369**	**(40.047, 6570.994)**	** < 0.001**	**440.321**	**(22.088, 8777.821)**	** < 0.001**
Offset ratio (CRVT/ASCO)	0.758	(0.435, 1.321)	0.328			
Glaucoma	0.888	(0.341, 2.310)	0.808			

Statistically significant values (*P* < 0.05) are shown in bold.

OR, Odds ratio; CI, Confidence interval; IOP, Intraocular pressure; BMO, Bruch’s membrane opening; ASCO, Anterior scleral canal opening.

*Variables with *P* < 0.10 in the univariable analysis were included in the subsequent multivariable analysis.

## Discussion

In this study, we discovered that the ASCO center was deviated from the BMO center and that this offset was closely associated with the CRVT position in both directionality and extent. From the BMO center, the ASCO offset was aligned in the direction of the CRVT offset, but to a lesser extent. The difference between the ASCO and CRVT offsets was the greatest in the inferior direction: albeit rarely, the CRVT was found to be deviated inferiorly from the BMO center, while the ASCO was not deviated inferiorly but was located in the vicinity of the BMO even in these cases. Furthermore, the demarcation of the ASCO margin was incomplete in 20% of eyes with the most severe misalignment, which suggests the limited value of the ASCO as a means of defining the offset between layers in such eyes.

The concept of ASCO/BMO offset was recently introduced to address the issue of the neural canal direction^23,24^. Using the Glaucoma Module Premium Edition of Spectralis machine, which was designed to visualize and demarcate the BMO preferentially, the BMO and ASCO margins were demarcated, and a plane was fitted for each layer. After projecting all of the points to the plane, a centroid was determined to define (1) the ASCO/BMO centroid vector, which connects each centroid, (2) the ASCO/BMO offset, a projection of the ASCO/BMO centroid vector to the BMO-fitted plane, and (3) neural canal obliqueness, the angle between the normal vector of the BMO-fitted plane and the ASCO/BMO centroid vector^23,24^. These parameters represented the neural canal direction, which has been reported to be associated with peripapillary retinal nerve fiber layer (RNFL) thickness distribution in healthy eyes^23^ and with ONH morphology in high myopia^24^.

Among the above-listed parameters, the ASCO/BMO offset signifies misalignment between the scleral and retinal layers^1–4^. This misalignment is thought to be acquired during eyeball expansion in the growth period^14–16^. The ONH morphology of newborns has been reported to be homogenous with the centrally located CRVT^21,22^. In the Boramae Myopia Cohort Study, serial examinations revealed actual shifting of the CRVT position prospectively^14–16^. Positional change of the CRVT is made possible by shifting of the surrounding LC and sclera^14–16^. In a subsequent 3D-MRI study, the direction of shift was closely associated with the eyeball shape, which supports our speculation that the diverse modes of eyeball expansion would result in diverse directionalities of misalignment between the scleral and retinal layers^17^. The ASCO, as the opening of the scleral layer at the ONH, would also be affected by misalignment between the scleral and retinal layers, except for one difference: the ASCO is located in a more superficial layer than the LC is. This difference of layer in the ONH canal could explain why, in the present study, the ASCO/BMO offset direction was more associated with the direction of the longest EOB than with that of the CRVT offset in the deeper layer.

**Figure 7:**
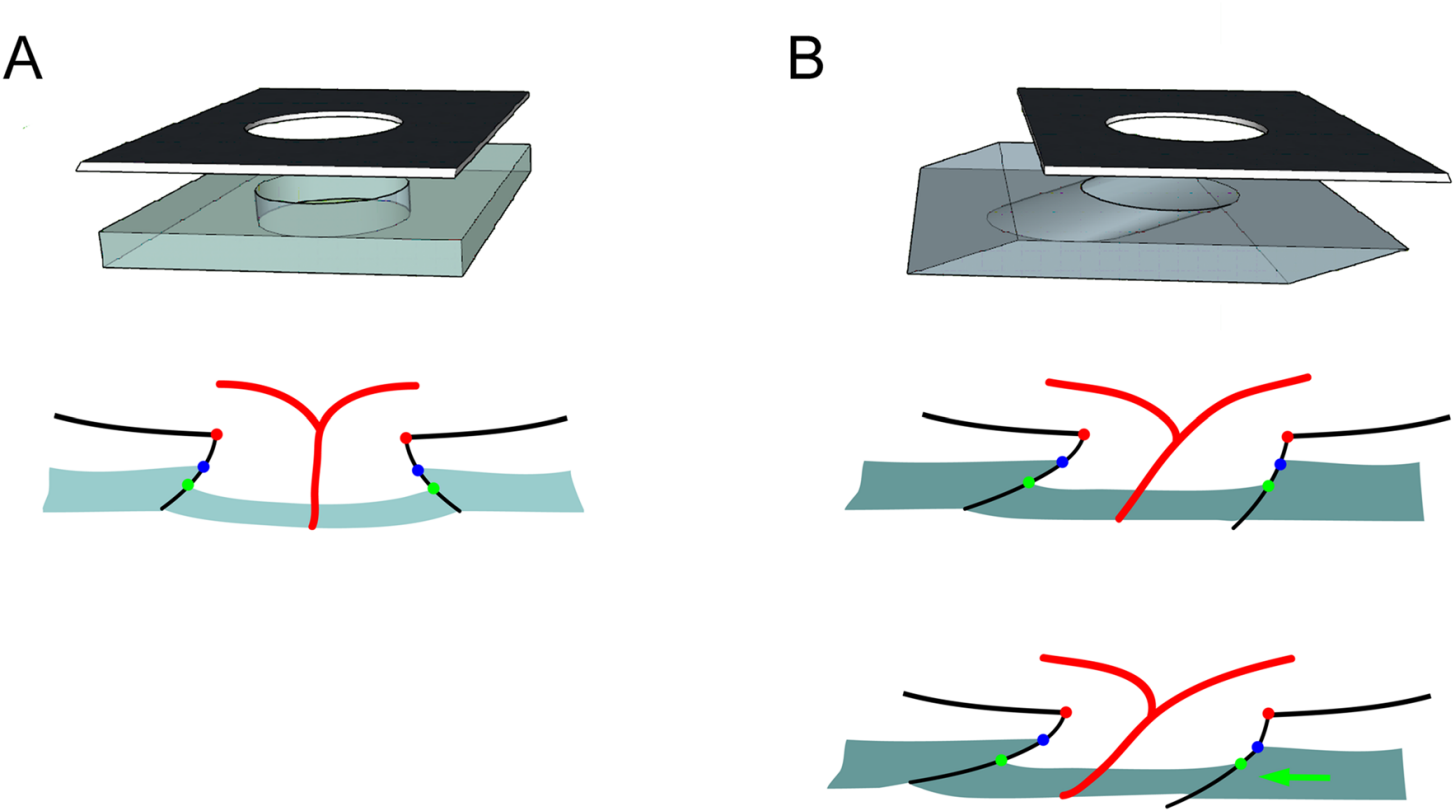
Schematic illustration of optic nerve head (ONH) canal in absence (**A**) and presence (**B**) of outer-wall shift. The intervening choroid is omitted for clarity. (Top) Three-dimensional view. A black overlying plane indicates the Bruch’s membrane opening (BMO), and the underneath cuboid with central hole indicates the scleral part of the ONH canal with the anterior scleral opening (ASCO) on the surface. (Bottom) Cross-sectional view. The red dots indicate the BMO margin, the blue dots the ASCO margin, the green dots the anterior lamina cribrosa (LC) insertion site, and the red lines the central retinal vascular trunk (CRVT). The peripapillary border tissue of the choroid (PBT-C) is the black line between the red and blue dots, and the peripapillary border tissue of the scleral flange (PBT-S) is the black line between the blue and green dots. The ASCO/BMO offset is the misalignment between the red and blue dots, and the LC/BMO offset, between the red and green dots. As the LC is located in a lower part of the ONH canal than the ASCO is, the LC/BMO offset is generally larger than the ASCO/BMO offset if the PBT-C and the PBT-S share a common directionality (**B**, Middle). If the PBT-S rotates further than the PBT-C, however, the LC/BMO offset can increase independently of the ASCO/BMO offset (**B**, Bottom; green arrow). We speculated that this could happen, due to the fact that the upper layer (the posterior polar retinal structure) is selectively affected by the restriction against expansion, whereas the lower layer (sclera and LC) is not.

The extent of shift was smaller for the ASCO/BMO offset than for the CRVT/BMO offset in the present study. This fact was in line with our previous study result, which revealed a larger change of CRVT position than that of γ-zone PPA during axial elongation^16^. The explanation is twofold. First, as stated above, the LC and ASCO are not on the same level: the LC is located below the ASCO. Therefore, the extent of LC offset would be greater than that of ASCO offset on the enface plane even if the ONH canal is just one tube with singular obliquity (Fig. 7). Second, the obliquity of the ONH canal may vary according to its depth. The lateral wall of the ONH canal consists of (1) the peripapillary border tissue of the choroid (PBT-C) and (2) the peripapillary border tissue of the scleral flange (PBT-S)^28^, and the PBT-C and the PBT-S can have different obliquity. This discrepancy might be exaggerated if the upper layer, which is closer to the retinal layer, is selectively affected by the restriction against expansion to preserve the posterior polar retinal structure. In that case, the PBT-S can rotate further than the PBT-C, and thereby the LC/BMO offset can surpass the ASCO/BMO offset (Fig. 7B).

**Figure 8:**
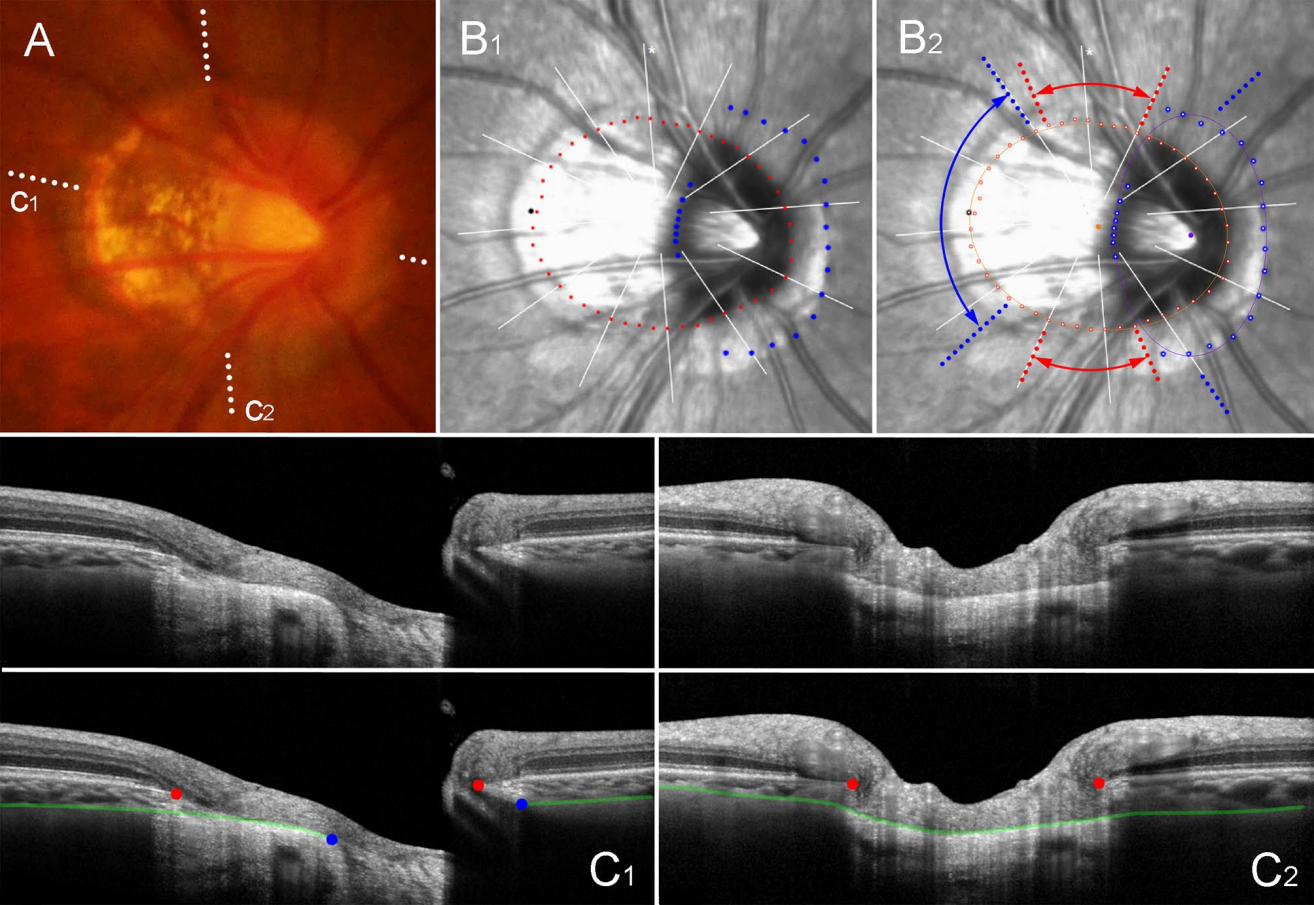
Critical disadvantage of determining anterior scleral opening (ASCO) based on radial scans centered on Bruch’s membrane opening (BMO) (right eye of 55 year-old-man without glaucoma, axial length of 25.50 mm). (**A**) Disc photograph. The dotted lines indicate the location of the OCT scan. (**B**) Infrared images. (**B**_**1**_) Bruch’s membrane opening (BMO, red dots), anterior scleral opening (ASCO, blue dots), and meridian of longest externally oblique border (EOB, black dot) are marked. (**B**_**2**_) Best-fitted ellipses for BMO (orange ellipse) and ASCO (purple ellipse) are drawn with their centers (orange dot for BMO center, purple dot for ASCO center). (**C**) B-scan OCT images. (top row: original images, bottom row: images with labels). The red dots indicate the BMO margin; the blue dots indicate the ASCO margin; the green lines indicate the anterior scleral surface. Please note that the B-scans between the red-dotted lines (**B**_**2**_) cannot yield any information about the ASCO, because they simply cannot capture the optic nerve canal at all (**C**_**2**_). Also, the B-scans between the blue-dotted lines (**B**_**2**_) produce uneven sampling for the ASCO: the ASCO margin closer to the BMO center (orange dot) is clustered within a very limited range (**B**_**2**_). Therefore, the ASCO itself is largely influenced by the ASCO/BMO offset, because no radial scans can focus on two different centers simultaneously. In this context, the ASCO/BMO offset as defined by BMO-oriented radial scans has irreparable disadvantage.

A sticking point, however, was that complete demarcation of the ASCO margin was not possible in 20% of cases. This might be attributable to the fact that the ASCO is a less distinctive anatomical structure than the BMO is. As in cases with scleral lip, the interface between the sclera and the stroma of choroid was not clearly distinguishable^29^. That was the reason why we could not project the anterior scleral surface plane through the ONH canal to obtain the ASCO as in previous studies^30,31^. More puzzling was the fact that incomplete demarcation of the ASCO margin was associated with larger ASCO/BMO offset after adjusting for covariables. This means that ASCO demarcation, which has been referenced to measure the ASCO/BMO offset, may itself be systematically affected by offset. The reason of such systematic bias is uneven sampling for the ASCO if the scan circle is centered to obtain even sampling for the BMO in the presence of offset (Fig. 8). Due to the offset, a range of radial scans (between the figure’s red arrows) did not yield any information about the ASCO, because the radial lines could not capture the ASCO in that range (Figs. 8B2 & C2). Even for the scans within the range for capturing the ASCO (between the blue arrows), the dots of the ASCO margin were unevenly sampled: there was clustered sampling near the BMO center (Fig. 8B2). To summarize, radial scans of the Glaucoma Module Premium Edition of the Spectralis OCT, which are best-fitted for the BMO, are rather biased for the ASCO, which bias tends to increase with the increase of the ASCO/BMO offset.

To handle incomplete and uneven sampling during demarcation, we used an ellipse-fitting method to define the center of dots (Supplemental Fig. 1). When a missing part exists, a geometric center is over-fitted to the observed points only, and the information about the missing part is simply lost. Loss of this information, however, produces a center biased toward non-missing points (Supplemental Fig. 1A). Uneven sampling could be understood as having missing points in a coarsely sampled area, and thereby has a similar effect. Although the ellipse-fitting method is based on the intuitive premise that the BMO and ASCO are ellipses, we believe that this method is still the best that we have to define a center from each opening.

A remarkable point is that the ASCO/BMO offset was rarely oriented to the inferotemporal direction. Previous studies’ scatter plots have shown the same phenomenon^23,24^. Superiorly located γ-zone PPA was not observed as frequently as it was in the nasal, temporal, and inferior locations. In our previous 3D-MRI study, we hypothesized that the direction of scleral layer shifting is determined by the mode of eyeball expansion: horizontal shifting by prolate/oblate growth and vertical shifting by asymmetric scleral growth^17^. The vast majority of vertical shift was superior, and we speculated that this might have been due to an inferior location of the embryonic fissure^32^, which possibly predisposes largely asymmetric over-growth on the inferior side^17^. Interestingly, the ASCO center was not deviated inferiorly, not even in the few cases with inferiorly located CRVTs (Fig. 6). Since the ASCO and BMO were nearly overlapped in those cases, we hypothesized that the anterior scleral surface was more resistant to inferior shifting than the LC and surrounding sclera were. In this case, the obliquity of the ONH canal was determined not by the PBT-C but by the PBT-S (Fig. 7B). In such cases, the ASCO offset cannot represent the LC shift directly, whereas the inferior LC shift is actually associated with glaucomatous RNFL defect in the superior hemifield^18,19^. This discrepancy of obliquity in the ONH canal as dependent on the directionality of the offset requires an explanation.

**Figure 9:**
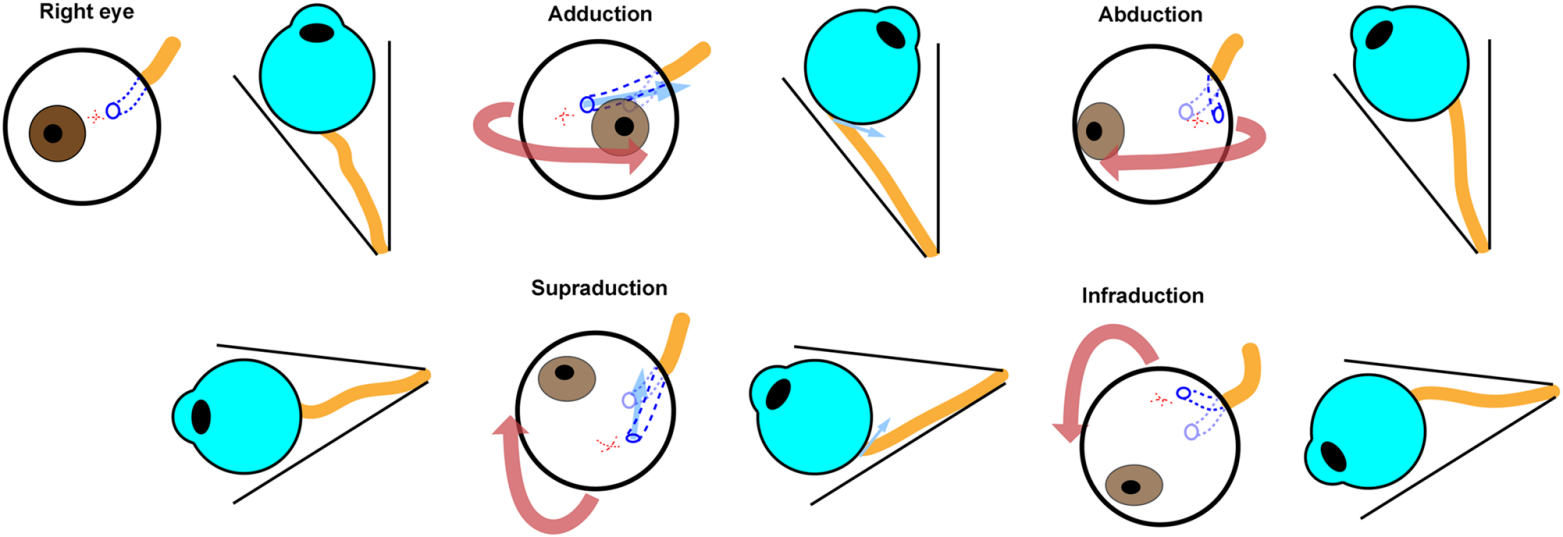
Tethering effect by optic nerve sheath during adduction, abduction, supraduction, and infraduction of right eye. During adduction, the anterior part of the eyeball is rotated nasally, which results in the rotation of the posterior pole (the dashed red cross) temporally (the red curved arrow) and subsequent traction to the scleral part of the ONH nasally (the blue arrow). Since the optic nerve sheath is attached to the posterior sclera, this effect will be larger in the deeper scleral part (the LC) than in the superficial part (the ASCO) of the ONH canal. Distally, the optic nerve sheath is anchored to the optic canal, which is located in the nasal and superior sides from the eyeball. Therefore, this tethering effect will be greater during adduction (nasal traction) and supraduction (superior traction) with a straightened path.

Larger offset in the deeper ONH canal and the preference for the superonasal direction might be explained by the repetitive mechanical loading on the ocular globe via the optic nerve sheath^33^ (Fig. 9). Eye movements generate significant ONH strains^34^, which may act as a source of acquired scleral deformation^35^. Since the optic nerve sheath is attached to the outer side of the sclera, the applied forces would be greater in the deeper part–the LC than in the superficial part–of the ASCO. Further, this tethering effect may be exerted differently according to the gaze-direction, due to the superonasal location of the optic canal, where the optic nerve sheath is anchored distally, from the eyeball. Adduction induces a greater tethering effect than does abduction, because, after rotation, the ONH is transposed farther from the nasally located optic canal^33^. Likewise, the superior location of the optic canal might exert a stronger tethering effect during supraduction than during infraduction^36,37^. Since supraduction is linked to the corresponding inferior transposition of the posterior pole and ONH, it would result in a tethering effect in the superior direction. Therefore, the ONH position may induce more tethering force during adduction and supraduction, which are linked to nasal and superior shift of the scleral layer, respectively (Fig. 9). Taken together, the scleral deformation induced by optic nerve sheath tethering would be larger in the deeper layer, and in the nasal and superior directions. Further study is required to confirm our speculation.

When the ASCO is located on the nasal side of the BMO, we can interpret this phenomenon interchangeably as nasal shift of the ASCO or temporal shift of the BMO; however, we prefer the former, for several reasons. First, the reference point for the measurement is the BMO. The Glaucoma Module Premium Edition of Spectralis is designed to detect the BMO initially, and so radial scans are centered on the BMO. As we stated above, as the radial scans become more fitted to the BMO center, they become more skewed from the ASCO center if there is an offset. Therefore, the ASCO is not as solid a parameter for reference-point purposes as the BMO is. Second, describing the ‘shift’ of the BMO can give a false impression of the BMO as approaching to the fovea, which is not the case at all. Preferential lengthening of the BM and retina may occur in the equatorial region circumferentially, which moves the BMO and fovea as a whole posteriorly along the *z*-axis^17^. So, the BMO itself is not shifted in any direction on the enface plane; rather, the posterior pole including the BMO just moves posteriorly with axial elongation. We supposed that perseveration of the posterior polar retinal structure would be significant, since its cellular density is directly related to resolution or visual acuity. This supposition is supported by the non-decrease of the retinal pigment epithelial cell density in the posterior pole with axial elongation, in contrast to its decrease in other areas^38^. If the posterior polar retinal structure is a stable segment as a whole, the change should be described based on it as a reference. Third, as we stated above, the sclera itself can be dragged by the optic nerve sheath tethering^33,34^. With regard to this force, the term ‘BMO shift’ is misleading, because the applied force and actual shift occurs in the sclera, not in the BMO.

The offset of openings signifies how twisted the paths of the RGCs are, since their cell bodies are anchored to the retinal plane while their axons pass through the misaligned outer wall to exit the eyeball. Therefore, the offset of openings will have clinical significance when evaluating disease affecting RGCs such as glaucoma. The offset was larger in glaucoma eyes than in their fellow control eyes in unilateral glaucoma^39^. The glaucomatous optic neuropathy occurred more frequently^18^ and earlier^19^ in the opposite direction of the LC offset. The offset of openings, however, is not a disease-specific extraordinary finding. Rather, it is ubiquitous change that can be observed in any eye if two premises are fulfilled during eyeball expansion: (1) preservation of the posterior polar retinal structure, and (2) scleral expansion during growth. Since the average axial length of newborns is around 17 mm, which is about 7 mm shorter than that of adults^40^, all eyes face their expansion period and are possibly subject to the offset. Although the current offset markers (CRVT position or ASCO/BMO offset) have their own limitations, complementary use of them may unveil the meaning of common but important anatomic change of the ONH in the whole population in the future.

This study has several limitations. First, our method of defining the ASCO center was slightly different from the previous works^30,31^ and should be interpreted with caution. Instead of projecting the anterior scleral surface plane to the ONH canal^30,31^, we left the ASCO unmarked if the anterior scleral surface was not traceable at the ONH canal and used the ellipse fitting method to define a center in the presence of missing values. Any method based on radial scanning centered on the BMO center, however, is subject to systemic bias, as already explained. A better method to address the problem of ASCO/BMO offset is required. Second, the CRVT position was used as a surrogate of LC offset. If we could completely demarcate the LC insertion site, we might have a better surrogate of LC offset. The LC insertion margin, however, was not clearly visible, due to the outward slope of the ONH canal in general. Further, the presence of offset rendered demarcation of the LC insertion margin more complicated. Third, all of the participants were South Korean, and there may be ethnic differences in ONH morphology^41^. Further research may be required to validate the current findings for different populations. Fourth, both glaucoma patients and healthy subjects were included, implying a possible confounding effect of LC remodeling in glaucomatous eyes. To minimize such effect, we included only glaucoma patients of the early-to-moderate stage. Also, notably, the additional analyses for each group revealed the same results (Supplemental Tables 2–3). It should be noted in this regard that glaucomatous ONH change has been reported to not affect the CRVT position in the LC portion^19,42^; nonetheless, the effect of LC remodeling on the LC offset should be investigated in the future. Fifth and finally, the CRVT/BMO offset as well as the ASCO/BMO offset were defined on the enface plane, which definition did not account for information along the *z*-axis in the ONH canal. An option of addressing this issue in future work might be the use of parameters such as neural canal obliqueness^23,24^.

In conclusion, ASCO offset from the BMO shared the directionality of LC offset but to a lesser extent. Therefore, ASCO/BMO offset can be used as an indicator of offset between the retinal and scleral layers in the ONH only after careful consideration of whether the LC could be shifted further from the ASCO, and whether the ASCO margin as detected on BMO-centered radial scans could be biased.

## Methods

### Study participants

This investigation was based on subjects who had been enrolled in the Boramae Glaucoma Imaging Study (BGIS), an ongoing prospective study at Seoul National University Boramae Medical Center (Seoul, Korea)^17–19^. This study registered the anatomic features of the ONH in subjects who had visited our institution with either a diagnosis of glaucoma or suspicion of glaucoma. Written informed consent to participate was obtained from all of the subjects. The study protocol was approved by the Seoul National University Boramae Medical Center Institutional Review Board and conformed to the tenets of the Declaration of Helsinki.

Subjects who were enrolled in the BGIS underwent a full ophthalmologic examination that included best-corrected visual acuity (BCVA) assessment, refraction, slit-lamp biomicroscopy, Goldmann applanation tonometry, gonioscopy, dilated funduscopic examination, keratometry (RKT-7700; Nidek, Hiroshi, Japan), axial length measurement (IOLMaster version 5; Carl Zeiss Meditec, Dublin, CA, USA), disc photography along with red-free fundus photography (TRC-NW8; Topcon, Tokyo, Japan), and SD-OCT (Spectralis OCT, Heidelberg Engineering, Heidelberg, Germany)^17–19^. The magnification error was adjusted by entering the corneal curvature of each eye into the SD-OCT system (Spectralis, Heidelberg Engineering) before scanning. During the acquisition of the SD-OCT images, the subjects were asked to fixate on a target, and images were acquired with the forehead and chin stabilized by the headrest. Extra care was taken during each exam to confirm that the forehead and chin were correctly positioned and did not move. Glaucomatous optic nerve damage was defined by rim thinning, notching, and the presence of RNFL defects, as evaluated by a glaucoma specialist (SHK). Glaucomatous visual field defect was defined as (1) outside normal limits on the glaucoma hemifield test; or (2) 3 abnormal points with a *P* value less than 5% probability of being normal, and 1 with a *P* value less than 1% by pattern deviation; or (3) a pattern standard deviation less than 5%, as confirmed on 2 consecutive reliable tests (fixation loss rate of ≤ 20%, and false-positive and false-negative error rates of ≤ 25%). Glaucoma was defined as glaucomatous optic nerve damage and associated visual field defects.

The inclusion criteria were healthy subjects, glaucoma-suspect or early-to-moderate glaucoma (mean deviation of ≥ -12.0 dB). The exclusion criteria were BCVA of < 20/40, poor-quality image (i.e., quality score < 15) of any section on enhanced depth imaging (EDI) SD-OCT radial scans, incomplete demarcation of BMO margin, incomplete demarcation of ASCO for more than 90° continuously and cases where the CRVT was located within the BMO but was impossible to determine clearly due to vessel bifurcation. If both eyes were eligible, one eye was randomly selected as the study eye.

### Demarcation of BMO and ASCO

**Figure 10:**
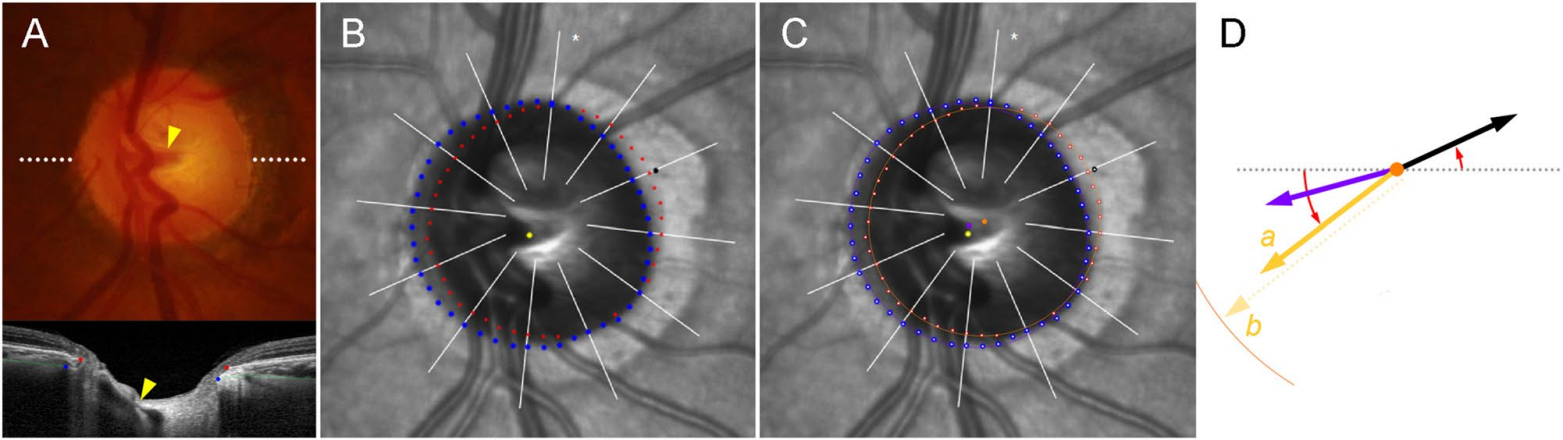
Determination of Bruch’s membrane opening (BMO), anterior scleral opening (ASCO), and central retinal vascular trunk (CRVT) (left eye of 65-year-old man without glaucoma, axial length of 23.72 mm). (**A**) Disc photograph and OCT image. The white dotted line indicates the location of the OCT scan, as targeted to the CRVT (arrowheads). The B-scan OCT image clearly shows the emergence of the CRVT. The red dots indicate the BMO margin. The ASCO margin (blue dots) was determined as the termination of the anterior scleral surface (green lines) at the optic nerve head canal. (**B**) Infrared image obtained by OCT. The margins of the BMO (red dots) and ASCO (blue dots) are demarcated. The CRVT position (yellow dot) and the meridian of the longest externally oblique border (EOB, black dot) are marked on the same infrared image. (**C**) Optimal ellipse fitting to obtain BMO and ASCO centers. Recognition of every point by our software is expressed by an inner white dot. The best-fitted ellipse for the BMO (orange ellipse) and the ASCO (purple ellipse) are drawn in reference to their centers (orange dot for BMO center, purple dot for ASCO center). (**D**) Calculation of offsets from given set of dots. From the BMO center (orange dot), the angular deviation of CRVT (yellow arrow), and ASCO center (purple arrow) are measured clockwise, with the nasal horizontal midline as 0°. A positive value indicates the superior location and a negative value indicates the inferior location. Likewise, the meridian of the longest EOB (black arrow) is measured clockwise, with the temporal horizontal midline as 0°. A positive value indicates the superior location and a negative value indicates the inferior location. From the BMO center, the distances are measured to the CRVT (*a*) and to the BMO margin in the same direction (*b*). The ratio of these distances is defined as the “shift index” (*a*/*b*), which is used to measure the extent of shift. The extent of CRVT offset (yellow arrow) divided by that of ASCO offset (purple arrow) is defined as the “offset ratio”.

BMO and ASCO were demarcated on infra-red images obtained by the Glaucoma Module Premium Edition of Spectralis OCT (Fig. 10). In this mode, the deep-ONH complex was imaged with 24 high-resolution radial scans taken in 15° increments. Each radial scan image was obtained by averaging of 24 individual B-scan images.

SD-OCT automatically detected the margin of the BMO. Every detected BMO margin was reviewed by one of the authors (KML), and errors were corrected manually (Fig. 10, red dots). On the same infra-red image, two independent observers (KML and MK) marked the ASCO margin manually by referring to corresponding cross-sectional B-scan images (Fig. 10, blue dots). The ASCO margin was defined as the termination of the anterior scleral surface, which was demarcated as the highly reflective border, at the ONH canal^30,31^. Thus, two points of the BMO as well as two points of the ASCO were demarcated along each radial scan. If any of the observers could not trace the anterior scleral surface to the point directly adjacent to the ONH canal, the ASCO was left unmarked along that radian. If the indiscernible angle was larger than 30° continuously, when measured from its center, that eye was classified as “incomplete demarcation for ASCO” and grouped accordingly.

### Assessment of offset between BMO and ASCO

To determine each center of BMO and ASCO respectively, we developed a MATLAB code that fits the best ellipse for a given set of dots. In brief, pixel values on the *x*–*y*-plane were obtained from all points (Fig. 10B). An ellipse was defined as a second-order polynomial on the *x*–*y*-plane: $a{x}^{2}+bxy+c{y}^{2}+dx+ey+f=0$. Based on the distances between the ellipse and the given set of dots, we defined the cost function, which is the squared sum of all of the distances. The optimal ellipse, which is to say, that which minimizes the cost function, was drawn using the Nelder-Mead method (Appendix). The center of the best-fitted ellipse was determined for the BMO and ASCO, and was defined as the BMO center and ASCO center, respectively. The offset of ASCO from BMO was calculated from the pixel values of each center (Fig. 10D, purple arrow).

For comparison, two other ONH parameters were marked on the same infrared image: 1) the CRVT position and 2) the meridian of the longest EOB. The CRVT position was measured from the BMO center as described previously^18,19^. Its emergence was demarcated on fundoscopic infrared images and color-disc photography, and was confirmed by cross-sectional SD-OCT imaging in all cases (Fig. 10A, arrowheads). In cases with an invisible CRVT on infrared fundus photographs and B-scan EDI SD-OCT images, either fluorescein or OCT angiography (Spectralis) was used to determine the presence of the CRVT within the BMO. The EOB was determined by the obliquity of the ONH borders on the radial B-scan images. The CRVT position and the meridian of the longest EOB (in cases larger than 100 µm) were marked on the infrared image, and their pixel values were also read by our customized software.

From the BMO center, the offset of each parameter was defined by angular deviation and extent. For the ASCO center and CRVT position, the angular deviation was measured based on the right-eye orientation, with the nasal horizontal midline as 0° (a positive value indicative of superior location, and a negative value indicative of inferior location)^18,19^. For the meridian of the longest EOB, the angular deviation was measured in the same way except that the temporal horizontal midline was 0° (Fig. 10D)^18,19^. To evaluate the extent of shift, the distance of the CRVT from the center of the BMO was (*a*) divided by the distance of the BMO margin from the center of the BMO in that direction and (*b*) defined as the “shift index” (Fig. 10D, a/b)^18,19^. In cases of invisible CRVT due to being located outside the BMO, the shift index was defined as 1.0, and the angular deviations were not determined. Those cases were excluded from the analysis of the angular deviation of the CRVT offset. The “offset ratio” was defined in order to compare the extent of offset quantitatively as the extent of CRVT offset divided by that of ASCO offset (Fig. 10D).

The BMO area was calculated by the Spectralis device, and the ASCO area was estimated by means of the ratio of the ASCO fitting ellipse area over the BMO fitting ellipse area. All the measurements were performed automatically by our customized software.

### Data analysis

The normal distribution of data was tested with the Shapiro–Wilk W test. The group comparisons were performed by way of the independent *t*-test or Mann–Whitney U test for the continuous variables and by chi-square testing for the categorical variables. Interobserver reproducibility for determination of the centers of the BMO & ASCO, the CRVT location, and the longest meridian of the EOB was evaluated by the pixel values of the *x* and *y* coordinates obtained by two independent observers (KML and MK) in 30 randomly selected images from 30 subjects. The standard deviation was calculated, and the coefficient of variation was estimated using, the one-way ANOVA random-effects model^25,26^. The inter-rater reliability of incomplete demarcation of ASCO was assessed by the Cohen’s kappa value. Logistic regression analysis was used to determine the factors associated with incomplete demarcation of ASCO. Univariable and multivariable analyses were used to determine the factors, and parameters with a *P* value less than 0.10 in the univariable analysis were included in the subsequent multivariable analysis. Statistical analyses were performed with commercially available software (Stata version 16.0; StataCorp, College Station, TX, USA) and R statistical packages version 3.4.3 (available at http://www.r-project.org; assessed December 5, 2017). The data herein are presented as mean ± standard deviations except where stated otherwise, and the cutoff for statistical significance was set to *P* < 0.05.

## Supplementary Information

Supplementary Information 1.

Supplementary Information 2.
