# Supplementary material for: Offset of openings in optic nerve head canal at level of Bruch’s membrane, anterior sclera, and lamina cribrosa
Source: Sci Rep. 2021 Nov 17;11:22435. doi: 10.1038/s41598-021-01184-8 (PMC8599705; doi:10.1038/s41598-021-01184-8)
Supplement: Supplementary file 1 — Supplementary Information 1. [file 41598_2021_1184_MOESM1_ESM.pdf]

Offset of openings in optic nerve head canal at level of Bruch's membrane, anterior sclera, and lamina cribrosa

Running Head: ASCO/BMO offset and LC shift

Kyoung Min Lee, M.D.[1,2], Hyoung Jun Ahn, Ph.D.[3], Martha Kim, M.D.[4], Sohee Oh, Ph.D.[5], and Seok Hwan Kim, M.D.[1,2]

[1]Department of Ophthalmology, Seoul National University College of Medicine, Seoul, Korea

[2]Department of Ophthalmology, Seoul National University Boramae Medical Center, Seoul, Korea

[3]Department of Mathematical modeling, Mind Flow Lab, Seoul, Korea

[4]Department of Ophthalmology, Dongguk University Ilsan Hospital, Goyang, Korea

[5]Department of Biostatistics, Seoul National University Boramae Medical Center, Seoul, Korea

Correspondence to: Seok Hwan Kim

Department of Ophthalmology, Seoul National University Boramae Medical Center, 39 Boramae Road, Dongjak-gu, 07061, Seoul, Korea

Tel: 82-02-870-2415, Fax: 82-02-831-2826, e-mail: xcski@hanmail.net

Financial Support: This work was supported by the KIST Institutional Program (2E30140). The funders had no role in the study design, data collection and analysis, decision to publish, or preparation of the manuscript.

Supplemental Figure 1. (**A**) Effect of missing values on ellipse-fitting method and centroid (arithmetic mean position of all points). To compare the effect of missing values between the ellipse-fitting method and the centroid, we arbitrarily deleted the continuous dots (the X-marked hollow dots) from the given set of dots (the red filled dots): the anterior scleral opening (ASCO) of Fig. 1. ($\mathbf{A_1}$) Complete demarcation, ($\mathbf{A_2}$) deleting 3 dots, ($\mathbf{A_3}$) deleting 6 dots. ($\mathbf{A_1}$) The best-fitted ellipse for all the dots was fitted, and its center is marked as a purple dot. A centroid for all the dots was simply calculated by averaging and is marked as a green dot. ($\mathbf{A_2}$, $\mathbf{A_3}$) In the cases with missing values. The best-fitted ellipse for the given set of dots (without hollow dots; red ellipse) was fitted, and its center is marked as a red dot. A centroid for the same set of dots was simply calculated by averaging and is marked as a blue dot. For comparison, the best-fitted ellipse, ellipse center, and centroid using all the dots are marked with the purple ellipse, the purple dot, and the green dot, respectively. From the original center, the angular range of the incomplete demarcation (between the grey arrows) was measured as 34° ($\mathbf{A_2}$) and 58° ($\mathbf{A_3}$). To visualize the difference between the dots, central areas are magnified (middle and right column). Even in a case of complete demarcation ($\mathbf{A_1}$), the center of the ellipse and centroid are different because the radial scans were not centered on the ASCO when they were obtained initially. Therefore, the centroid is subject to the uneven sampling despite the complete demarcation. Such a difference is amplified by the incomplete demarcation since the centroid is overfitted to the observed points only ($\mathbf{A_2}$ and $\mathbf{A_3}$). Despite the increase of the indiscernible angle, the center of the best-fitted ellipse is nearly fixed, in contrast to the increased discrepancy of the centroid (between the blue arrows). The *x* and *y* axis values are in pixels. (**B**) Range of best ellipse depending on degree of indiscernible angle. To simulate the variability of the ellipse-fitting method as dependent on the demarcation status, we arbitrarily deleted the continuous dots (the hollow dots) from the given set of dots (the red filled dots): the anterior scleral opening (ASCO) of Fig. 1. ($\mathbf{B_1}$) Complete demarcation, ($\mathbf{B_2}$) deleting 6 dots, ($\mathbf{B_3}$) deleting 10 dots, ($\mathbf{B_4}$) deleting 16 dots. Then, the best-fitted ellipse for the original entire set of dots (the purple ellipse), and the best-fitted ellipse for the given set of dots (without hollow dots; the red ellipse) were fitted using our customized code. For the same given set of dots, we also drew 'the most deviated second-best-fitted ellipse' (the blue ellipse). Numerically, the best-fitted ellipse was defined as the ellipse that minimizes its cost function: the squared sum of the distance between every dot from the given ellipse. To define 'the most deviated second-best-fitted ellipse', we found the most deviated ellipse while the increase of the cost-function was bounded, which stands for the limit of the deviation of the best-

fitted ellipse. At the center, the purple dot indicates the original center (without missing values), the red dot indicates the measured center (with missing values), and the blue dot indicates the center of the most deviated second-best-fitted ellipse (with missing values). Please note that the purple dots are invisible in (**B$_1$**) and (**B$_2$**) because they are overlapped by the red dots. From the original center (the purple dot), the angular range of the incomplete demarcation (between the grey arrows) was measured as 57° (**B$_2$**), 87° (**B$_3$**), and 130° (**B$_4$**). The possible range of the best-fitted ellipse (the red ellipse)'s center is drawn in thick solid-red lines. Please note that the range of the best-fitted ellipse center (the thick solid-red closed circuit at the center) was negligible until the indiscernible angle approached 90°. The *x* and *y* axis values are in pixels.

Supplemental Table 1. Interobserver reproducibility of determination of Bruch's membrane opening (BMO), anterior scleral opening (ASCO), central retinal vascular trunk (CRVT), and meridian of longest externally oblique border (EOB)

	BMO center		ASCO center		CRVT		Meridian of longest EOB	
	x-axis	*y*-axis	*x*-axis	*y*-axis	*x*-axis	*y*-axis	*x*-axis	*y*-axis
SD of difference (pixel)	0.734	0.530	1.439	1.198	1.829	1.490	2.659	2.087
CV (%)*	0.226	0.166	0.459	0.409	0.618	0.456	1.514	0.755

*CV was calculated using the one-way ANOVA random effects model

BMO = Bruch's membrane opening; ASCO = Anterior scleral canal opening; CRVT = Central retinal vascular trunk; EOB = Externally oblique border; SD = Standard deviation; CV = Coefficient of variation

Supplemental Table 2. Factors associated with angular deviation of anterior scleral opening (ASCO) offset in each group

	Univariable analysis			Multivariable analysis*		
Control	Coefficient	95% CI	*P*	Coefficient	95% CI	*P*
Age*, years*	0.914	(0.018, 1.810)	0.046	-0.222	(-0.619, 0.175)	0.265
Female (vs. male sex)	35.832	(12.215, 59.450)	0.004	-0.729	(-10.200, 11.658)	0.893
Axial length, *mm*	-14.902	(-23.357, -6.447)	0.001	-2.480	(-6.824, 1.863)	0.255
IOP, *mmHg*	-3.960	(-10.040, 2.121)	0.197			
BMO area, mm^2	-10.376	(-21.900, 1.148)	0.077	4.277	(-0.533, 9.088)	0.080
Foveal-BMO axis, °	-3.779	(-7.149, -0.409)	0.029	-1.039	(-2.517, 0.439)	0.163
ASCO area, mm^2	-16.224	(-35.718, 3.270)	0.101			
CRVT offset, °	0.047	(-0.139, 0.233)	0.613			
**Meridian of longest EOB, °**	**-0.768**	**(-0.879, -0.657)**	**<0.001**	**-0.751**	**(-0.886, -0.615)**	**<0.001**
	Univariable analysis			Multivariable analysis*		
Glaucoma	Coefficient	95% CI	*P*	Coefficient	95% CI	*P*
Age*, years*	1.853	(0.926, 2.780)	<0.001	0.101	(-0.496, 0.698)	0.732
Female (vs. male sex)	42.188	(16.610, 67.765)	0.002	-8.123	(-22.592, 6.346)	0.262
**Axial length, *mm***	**-13.855**	**(-21.432, -6.279)**	**0.001**	**-4.991**	**(-9.915, -0.066)**	**0.047**
IOP, *mmHg*	-2.667	(-8.135, 2.802)	0.332			
BMO area, mm^2	-31.591	(-55.581, -7.602)	0.011	-2.860	(-14.622, 8.902)	0.624

Foveal-BMO axis, °	-2.457	(-5.359, 0.446)	0.095	-0.648	(-1.833, 0.537)	0.274
ASCO area, *mm*^{2}	-21.019	(-47.435, 5.396)	0.116	-4.780	(-14.113, 4.553)	0.311
CRVT offset, °	0.372	(0.215, 0.528)	<0.001	0.058	(-0.031, 0.147)	0.195
**Meridian of longest EOB, °**	**-0.822**	**(-0.905, -0.739)**	**<0.001**	**-0.712**	**(-0.850, -0.574)**	**<0.001**

	Univariable analysis			Multivariable analysis*		
Myopia (Axial length ≥ 24mm)	Coefficient	95% CI	*P*	Coefficient	95% CI	*P*
Age*, years*	0.777	(0.081, 1.472)	0.029	-0.001	(-0.270, 0.269)	0.996
Female (vs. male sex)	**24.047**	**(4.691, 43.404)**	**0.016**	**-9.036**	**(-16.960, -1.111)**	**0.026**
**Axial length, *mm***	**-10.588**	**(-18.966, -2.210)**	**0.014**	**-3.338**	**(-6.532, -0.144)**	**0.041**
IOP, *mmHg*	-1.030	(-4.862, 2.803)	0.594			
BMO area, *mm*^{2}	-6.860	(-17.358, 3.639)	0.197			
Foveal-BMO axis, °	-2.691	(-4.738, -0.643)	0.011	-0.503	(-1.263, 0.256)	0.189
ASCO area, *mm*^{2}	-6.762	(-23.004, 9.481)	0.409			
CRVT offset, °	**0.397**	**(0.251, 0.542)**	**<0.001**	**0.352**	**(0.243, 0.462)**	**<0.001**
**Meridian of longest EOB, °**	**-0.769**	**(-0.857, -0.680)**	**<0.001**	**-0.428**	**(-0.552, -0.305)**	**<0.001**

CI = Confidence interval; IOP = Intraocular pressure; BMO = Bruch's membrane opening; ASCO = Anterior scleral canal opening; CRVT = Central retinal vascular trunk; EOB = Externally oblique border

Statistically significant values ($P < 0.05$) are shown in bold. *Variables with $P < 0.10$ in the univariable analysis were included in the subsequent multivariable analysis.

Supplemental Table 3. Risk factors for incomplete demarcation of anterior scleral opening (ASCO) margin in each group

	Univariable analysis			Multivariable analysis*		
Control	OR	95% CI	*P*	OR	95% CI	*P*
Age, *years*	0.938	(0.889, 0.991)	0.021	0.979	(0.917, 1.045)	0.527
Female (vs. male sex)	0.758	(0.200, 2.871)	0.683			
Axial length, *mm*	2.553	(1.321, 4.936)	0.005	1.500	(0.691, 3.257)	0.305
IOP, *mmHg*	0.973	(0.706, 1.339)	0.866			
BMO area, mm^2	1.725	(0.946, 3.145)	0.075	1.659	(0.777, 3.540)	0.191
Foveal-BMO axis, °	1.106	(0.910, 1.345)	0.312			
ASCO area, mm^2	1.847	(0.667, 5.112)	0.237			
**Shift Index**	**503.967**	**(13.458, 18871.6)**	**0.001**	**188.818**	**(2.811, 12684.32)**	**0.015**
Offset ratio (CRVT/ASCO)	0.831	(0.497, 1.391)	0.481			

	Univariable analysis			Multivariable analysis*		
Glaucoma	OR	95% CI	*P*	OR	95% CI	*P*
Age, *years*	0.908	(0.841, 0.981)	0.014	1.007	(0.908, 1.115)	0.901
Female (vs. male sex)	0.723	(0.163, 3.200)	0.669			
Axial length, *mm*	1.662	(0.987, 2.798)	0.056	1.420	(0.682, 2.956)	0.348
IOP, *mmHg*	1.254	(0.935, 1.681)	0.131			
BMO area, mm^2	2.390	(0.677, 8.438)	0.176			

Foveal-BMO axis, °	1.041	(0.880, 1.232)	0.640			
ASCO area, *mm*^{2}	0.906	(0.226, 3.638)	0.890			
**Shift Index**	**630.414**	**(16.854, 23579.58)**	**<0.001**	**625.668**	**(6.719, 58259.38)**	**0.005**
Offset ratio (CRVT/ASCO)	0.503	(0.130, 1.953)	0.321			
	Univariable analysis			Multivariable analysis*		
Myopia (Axial length ≥ 24mm)	OR	95% CI	*P*	OR	95% CI	*P*
Age, *years*	0.949	(0.907, 0.992)	0.022	0.982	(0.933, 1.034)	0.494
Female (vs. male sex)	1.245	(0.419, 3.704)	0.693			
Axial length, *mm*	1.646	(0.992, 2.733)	0.054	1.123	(0.605, 2.082)	0.714
IOP, *mmHg*	1.018	(0.824, 1.256)	0.871			
BMO area, *mm*^{2}	1.595	(0.878, 2.898)	0.125			
Foveal-BMO axis, °	1.081	(0.941, 1.242)	0.272			
ASCO area, *mm*^{2}	1.333	(0.548, 3.245)	0.526			
**Shift Index**	**211.626**	**(15.341, 2919.416)**	**<0.001**	**136.063**	**(8.272, 2238.089)**	**0.001**
Offset ratio (CRVT/ASCO)	0.822	(0.435, 1.555)	0.547			

OR = Odds ratio; CI = Confidence interval; IOP = Intraocular pressure; BMO = Bruch's membrane opening; ASCO = Anterior scleral canal opening; CRVT = Central retinal vascular trunk

Statistically significant values ($P < 0.05$) are shown in bold. *Variables with $P < 0.10$ in the univariable analysis were included in the subsequent multivariable analysis.

Appendix.

Let us represent a general conic by an implicit second order polynomial:

$$ax^2 + bxy + cy^2 + dx + ey + f = 0$$

We want to find the initial ellipse equation $(\hat{a}, \hat{b}, \hat{c}, \hat{d}, \hat{e}, \hat{f})$ that fits $(x_1, y_1), \cdots, (x_n, y_n)$ by

$$(\hat{a}, \hat{b}, \hat{c}, \hat{d}, \hat{e}, \hat{f}) = argmin \sum_{k=1}^{n} (ax_k^2 + bx_k y_k + cy_k^2 + dx_k + ey_k + f)^2$$

satisfying $(a^2 + b^2 + c^2 + d^2 + e^2 + f^2 = 1)$.

This equation can be solved by linear algebra. We note that the equation above is different from the rigorous definition of the cost function, since squared value of the implicit second-order polynomial is not equal to the squared value of the distance between the given ellipse and point (x_k, y_k).

Then, our goal is to find the best ellipse M that minimizes

$$\sum_{k=1}^{n} d\big(M, (x_k, y_k)\big)^2$$

where $d\big(M, (x_k, y_k)\big)$ is the distance between ellipse M and point (x_k, y_k).

The mathematical definition is as follows:

$$d\big(M, (x_k, y_k)\big) = \min_{(x,y)\in M} \sqrt{(x - x_k)^2 + (y - y_k)^2}.$$

This optimization problem is solved numerically (by the Nelder-Mead method).
