# Supplementary figures and images for: Offset of openings in optic nerve head canal at level of Bruch’s membrane, anterior sclera, and lamina cribrosa
Source: Sci Rep. 2021 Nov 17;11:22435. doi: 10.1038/s41598-021-01184-8 (PMC8599705; doi:10.1038/s41598-021-01184-8)

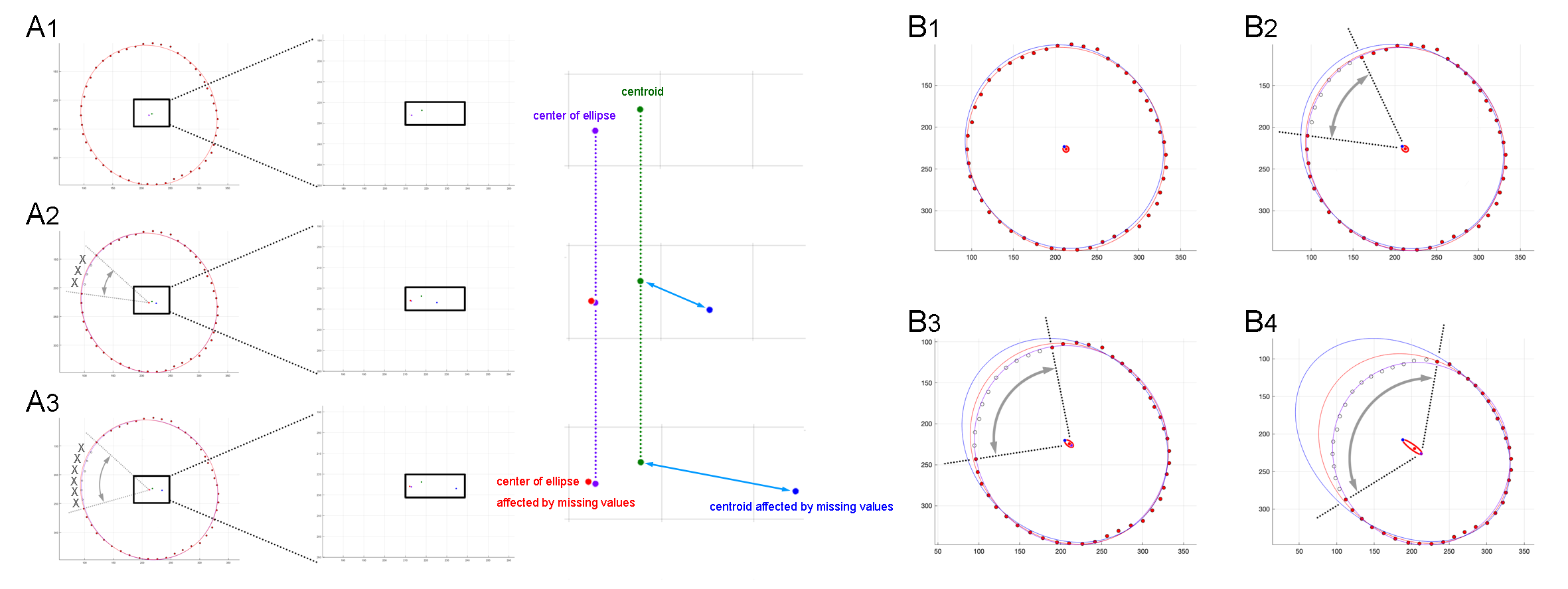

Supplement: Supplementary file 2 — Supplementary Information 2. [file 41598_2021_1184_MOESM2_ESM.tif]
